# Optimized pipeline of MuTect and GATK tools to improve the detection of somatic single nucleotide polymorphisms in whole-exome sequencing data

**DOI:** 10.1186/s12859-016-1190-7

**Published:** 2016-11-08

**Authors:** Ítalo Faria do Valle, Enrico Giampieri, Giorgia Simonetti, Antonella Padella, Marco Manfrini, Anna Ferrari, Cristina Papayannidis, Isabella Zironi, Marianna Garonzi, Simona Bernardi, Massimo Delledonne, Giovanni Martinelli, Daniel Remondini, Gastone Castellani

**Affiliations:** 1 Department of Physics and Astronomy, University of Bologna, Bologna, Italy; 2 CAPES Foundation, Ministry of Education of Brazil, Brasília, DF Brazil; 3 Department of Experimental, Diagnostic and Specialty Medicine, University of Bologna, Bologna, Italy; 4 Department of Biotechnology, University of Verona, Verona, Italy; 5 Unit of Blood Diseases and Stem Cell Transplantation, Department of Clinical and Experimental Sciences, University of Brescia, Brescia, Italy; 6 Personal Genomics, Verona, Italy

**Keywords:** Cancer, Somatic single nucleotide variants, Whole exome sequencing

## Abstract

**Background:**

Detecting somatic mutations in whole exome sequencing data of cancer samples has become a popular approach for profiling cancer development, progression and chemotherapy resistance. Several studies have proposed software packages, filters and parametrizations. However, many research groups reported low concordance among different methods. We aimed to develop a pipeline which detects a wide range of single nucleotide mutations with high validation rates. We combined two standard tools – Genome Analysis Toolkit (GATK) and MuTect – to create the GATK-LOD_N_ method. As proof of principle, we applied our pipeline to exome sequencing data of hematological (Acute Myeloid and Acute Lymphoblastic Leukemias) and solid (Gastrointestinal Stromal Tumor and Lung Adenocarcinoma) tumors. We performed experiments on simulated data to test the sensitivity and specificity of our pipeline.

**Results:**

The software MuTect presented the highest validation rate (90 %) for mutation detection, but limited number of somatic mutations detected. The GATK detected a high number of mutations but with low specificity. The GATK-LOD_N_ increased the performance of the GATK variant detection (from 5 of 14 to 3 of 4 confirmed variants), while preserving mutations not detected by MuTect. However, GATK-LOD_N_ filtered more variants in the hematological samples than in the solid tumors. Experiments in simulated data demonstrated that GATK-LOD_N_ increased both specificity and sensitivity of GATK results.

**Conclusion:**

We presented a pipeline that detects a wide range of somatic single nucleotide variants, with good validation rates, from exome sequencing data of cancer samples. We also showed the advantage of combining standard algorithms to create the GATK-LOD_N_ method, that increased specificity and sensitivity of GATK results. This pipeline can be helpful in discovery studies aimed to profile the somatic mutational landscape of cancer genomes.

**Electronic supplementary material:**

The online version of this article (doi:10.1186/s12859-016-1190-7) contains supplementary material, which is available to authorized users.

## Background

Somatic mutations play a key role in cancer development, progression and chemotherapy resistance. Therefore, several studies have been profiling somatic mutations in cancer samples by applying next generation sequencing technologies, allowing the discovery of drug targets, prognostic DNA markers and protocols of targeted therapies. Whole Exome Sequencing (WES) has become a popular approach because it is cost effective and it detects approximately 25,000 single nucleotide variants (SNVs) in the coding region of human DNA. However, the detection of somatic mutations in normal-cancer paired samples presents unique challenges: 1) detecting low allelic frequency mutations due to tumor heterogeneity, subclonality and copy number variation events; 2) differentiating true mutations from alignment artifacts and sequencing errors; 3) classifying mutations as somatic or germ-line polymorphisms; and 4) analyzing tumor samples contaminated by normal cells and vice-versa [1]. The understanding of the mutational landscape of cancer genomes requires the development of methods that detect somatic mutations and deal with these challenges.

Several studies have compared the performance of different pipelines, softwares and parametrizations [2–7]. In general, the available tools classify the somatic mutations by either independently or simultaneously analyzing the tumor and normal samples; but, since they have different prior assumptions and error modeling approaches, many research groups have reported low concordance among methods [4, 8]. The available tools either detect too many false positives in order to get all true positives or lose too many true positives in order to reduce the number of false positives [9]. In the first case, the researcher spends much time and resource validating the set of candidate variants to select the true ones. In the second case, important mutations that explain the biological characteristics of the cancer cells, may be missed. This evidence, along with the variability in the performance of each software according to studies and tumor type, indicates that the research community faces a big challenge choosing the right pipeline among all available options.

In this study, we aimed to develop a pipeline that detects a wide and high confident profile of single nucleotide variants in sequencing data of cancer samples. Our pipeline brings together the benefits of two standard tools: Genome Analysis Toolkit (GATK) and MuTect. GATK independently calls variants in the normal and tumor samples, while MuTect performs the analysis simultaneously. We created the GATK-LOD_N_ method, which is part of the MuTect algorithm, that is applied downstream to the GATK analysis in order to ensure the somatic classification of the GATK results and reduce its false positive calls. As proof of principle, we applied our pipeline to hematological (Acute Myeloid and Acute Lymphoblastic Leukemias) and solid (Gastrointestinal Stromal Tumor and Lung Adenocarcinoma) tumors. We also tested our pipeline on simulated data and technical replicate samples to evaluate its sensitivity and specificity. Our results show that the pipeline performed well and we believe that it can be helpful in discovery studies aimed to profile the somatic mutational landscape of cancer genomes.

## Methods

### Sequencing data

Primary samples were collected from Acute Myeloid Leukemia (*n* = 37) and Acute Lymphoblastic Leukemia patients (*n* = 41) after obtaining informed consent as approved by the Institutional Ethical Committee (protocol number 253/2013/O/Tess) of Azienda Ospedaliero-Universitaria, Policlinico Sant’Orsola-Malpighi (Bologna, Italy) in accordance with the Declaration of Helsinki. Leukocytes were enriched from bone marrow and peripheral blood samples by separation on Ficoll density gradient. Saliva samples, used as normal matching, were collected with the Oragene Discover kit (DNA Genotek). The DNA was extracted from leukocytes by column purification (AllPrep DNA/RNA/Protein Mini Kit and QIAcube, Qiagen) and from saliva by paramagnetic particles (Maxwell® 16 LEV DNA Blood Purification Kit and Maxwell® MDx Instrument), according to manufacturer’s protocol. The exonic regions were captured by TrueSeq™ Exome Enrichment Kit and Nextera Rapid Capture Expanded Exome, comprising a targeted region of 62 Mb, and 201,121 exonic regions. Illumina HiSeq2000 sequencing produced an average of 55.2 and 63 million 100 bp paired-end reads per sample in AML and ALL cohorts, respectively. The AML and ALL data sets are available upon request to the Next Generation Sequencing for Targeted Personalized Therapy of Leukemia consortium. We also selected two public datasets of Illumina HiSeq 2000 whole exome sequencing from the NCBI Sequence Read Archive: 1) seven Gastrointestinal Stromal Tumors (GIST) samples, and their matching peripheral blood samples, with an average of 35.5 million 100 bp paired-end reads per sample [SRA: SRR1299130-141 and SRR1299144-147] [10]; and 2) two Lung Adenocarcinoma samples, and their normal counterparts, with an average of 56.5 million 100 pb paired-end reads per sample [SRA: ERR160124, ERR160136, ERR166338, and ERR166339] [11]. After the quality control check, the average of final coverages were: 72X (±30X), 119X (±28X), 76X (±7X), 133X (±64X); for AML, ALL, GIST, and Lung Adenocarcinoma, respectively (Additional file 1 provides, for each tumor type, the samples IDs and coverage information).

### Pipeline for somatic variant discovery

Initially, the sequencing reads were submitted to a quality control check by using the scripts fastq_quality_filter.pl and fastq_quality_trimmer.pl from FASTX-Toolkit [12]. The phred value 20 was chosen as the minimum threshold for base quality. The reads having more than 80 % of low quality bases were removed or had their 3′ extremity bases trimmed when the minimum threshold was not reached. After, the reads were aligned to the human reference genome hg19/GRCh37 using BWA-MEM [13] with default parameters and Picard [14] was applied for post-alignment procedures as sorting, indexing, and marking duplicates. The alignments were submitted to local realignment around INDELs and base quality score recalibration (BQSR) by using the Genome Analysis Toolkit (GATK) version 3.0 [15].

MuTect [16] and GATK (Haplotype Caller) were used for the single nucleotide variant calling. GATK variants were filtered with the Variant Quality Score Recalibration tool following the best practices on the GATK website. GATK performs the variant calling and filtration in the normal and tumor samples independently, thus the subtraction between the tumor and the normal variants resulted in our first set of candidate somatic variants.

To ensure the somatic classification of the SNVs called by GATK, we adapted the MuTect algorithm and applied its LOD_N_ classifier after the GATK variant calling and filtering. The LOD_N_ is a bayesian classifier that compares the likelihood of two models: (1) the mutation does not exist in the normal sample and all non-reference bases are explained by sequencing noise, and (2) the mutation truly exists in the normal sample as a germ-line heterozygous variant. The ratio of these two likelihoods is called LOD (Log Odds) score and when it exceeds a decision threshold, the mutation can be classified as somatic. For this filtering, we considered only sites that had total read depth greater or equal than 8 in the normal sample and greater or equal than 14 in the tumor sample. Our final candidate list consisted in the union of MuTect and GATK-LOD_N_ results.

**Fig. 1:**
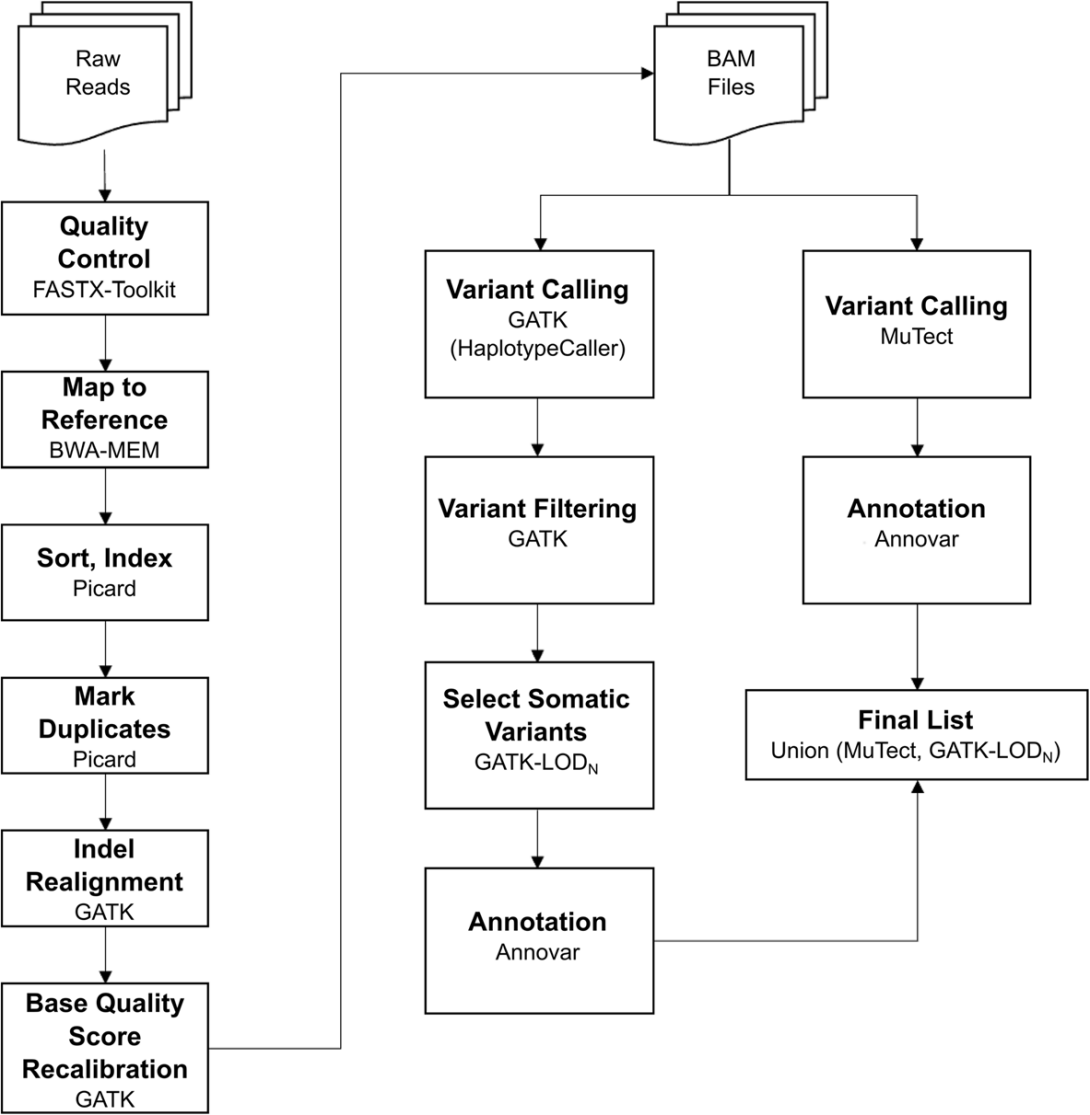
Pipeline of SNV detection in sequencing data of cancer samples. Summary of steps and their respective tools in the detection of SNVs in paired normal-cancer sequencing data

The variants were annotated by ANNOVAR [17], with the Ensembl Gene annotation database for human genome build 37 (http://www.ensembl.org/), and searched for matches in the dbSNP138 and 1000 Genomes data. We selected exonic single nucleotide variants (SNVs) that were non-synonymous and gain or loss of stop codon. Variants present in dbSNP138 and 1000 Genomes with minor allele frequency (MAF) greater than 0.05 were removed. Figure 1 shows the summary of the pipeline steps. The scripts for running the main pipeline steps are availabe in the link: https://bitbucket.org/BBDA-UNIBO/wes-pipeline.

A subset of variants from MuTect, GATK and GATK-LOD_N_ calls were selected for validation. Variants with allelic frequency higher than 0.2 were validated by Sanger Sequencing and those with allelic frequency lower than 0.2 were validated by using the Illumina TruSight Myeloid Sequencing Panel and Illumina MiSeq sequencing. Data were analyzed by the VariantStudio software (Illumina), according to manufacturer’s instruction.

### Pipeline testing

As MuTect eventually miscalled variants already profiled by Sanger sequencing at the moment of diagnosis, we tested adapting the MuTect algorithm by lowering its two main parameters and thresholds – Θ_T_ > = 6.5 and Θ_N|dbSNP site_ > = 5.5 – that determine the mutation detection and classification as somatic or germ-line. We calculated the Θ_T_ and Θ_N_ values for each variant in the GATK raw output and set the thresholds to the minimum values that would permit the correct classification of 10 variants previously identified by Sanger sequencing.

We simulated datasets to evaluate the specificity and sensitivity of the three variant calling methods: MuTect, GATK and GATK-LOD_N_. The specificity was evaluated by splitting the sequencing data of the same sample in two, applying the three variant calling methods, and counting the number of total SNVs called. One saliva sample of our AML cohort (80X) had its reads randomized (reads sorted by query name) and it was split in two by using the bamutils tool of NGSUtils package [18]. The resultant alignment files were applied to each variant calling method. The sensitivity was calculated by creating artificial tumor samples, applying the variant calling methods, and counting the number of true positives called. We adapted the mutate_sample.py script from the Shimmer package [19] to create mutations in a saliva sample alignment. Three artificial tumors were created with 22, 25 and 25 SNVs, which had variant allelic fractions range of 0.02 to 0.25, 0.5 to 0.86, and 0.97 to 1.0, respectively (Table 1). For each artificial tumor sample, we created subsets by randomly excluding reads and simulated sequencing coverages in the range of 5X to 80X, with intervals of 5X. The creation of the subsets was performed by the DownsampleBam tool of Picard. We then evaluated the performance of each variant calling method at different coverage levels.

**Table 1:** Artificial tumor samples. Coordinate list of the single nucleotide variants inserted in the artificial tumor samples and their variant allelic frequencies

Chromosome	Position	REF > ALT	Artificial tumors variant allelic frequencies			Normal variant allelic frequencies
			0.02 – 0.26	0.5 – 0.86	0.97 – 1	
11	19854088	G > A	0.03	0.69	1.00	0
11	36484167	C > T	0.08	0.62	1.00	0.027
11	4608116	T > C	0.13	0.71	1.00	0.020
11	4661826	T > C	0.11	0.60	0.97	0.028
11	4673788	G > A	0.26	0.64	1.00	0.021
11	4928841	T > C	0.13	0.61	1.00	0
11	5372856	A > G	0.24	0.69	1.00	0.023
11	5373562	C > A	0.09	0.68	1.00	0.029
11	5443887	T > C	0.10	0.86	1.00	0
11	5443893	G > A	0.10	0.86	1.00	0
11	5462255	C > G	0.16	0.56	1.00	0
11	5906203	T > G	0.19	0.70	1.00	0
11	6519642	G > A	0.08	0.61	1.00	0
11	824789	T > C	0.11	0.63	1.00	0.026
12	25398281	C > T	0.12	0.63	1.00	0
12	75715330	C > A	0.13	0.60	1.00	0
22	24891418	A > C	0.21	0.70	1.00	0.030
22	44083442	T > C	NA	0.78	1.00	0
13	101289801	C > A	0.13	0.65	1.00	0
20	61537337	G > T	0.13	0.65	1.00	0
17	48557299	G > T	0.11	0.74	1.00	0
5	45262378	G > T	0.08	0.50	1.00	0
1	94476902	T > C	0.15	0.65	1.00	0
2	110372199	G > T	NA	0.57	1.00	0
5	64907465	C > A	0.10	0.57	1.00	0

## Results

**Fig. 2:**
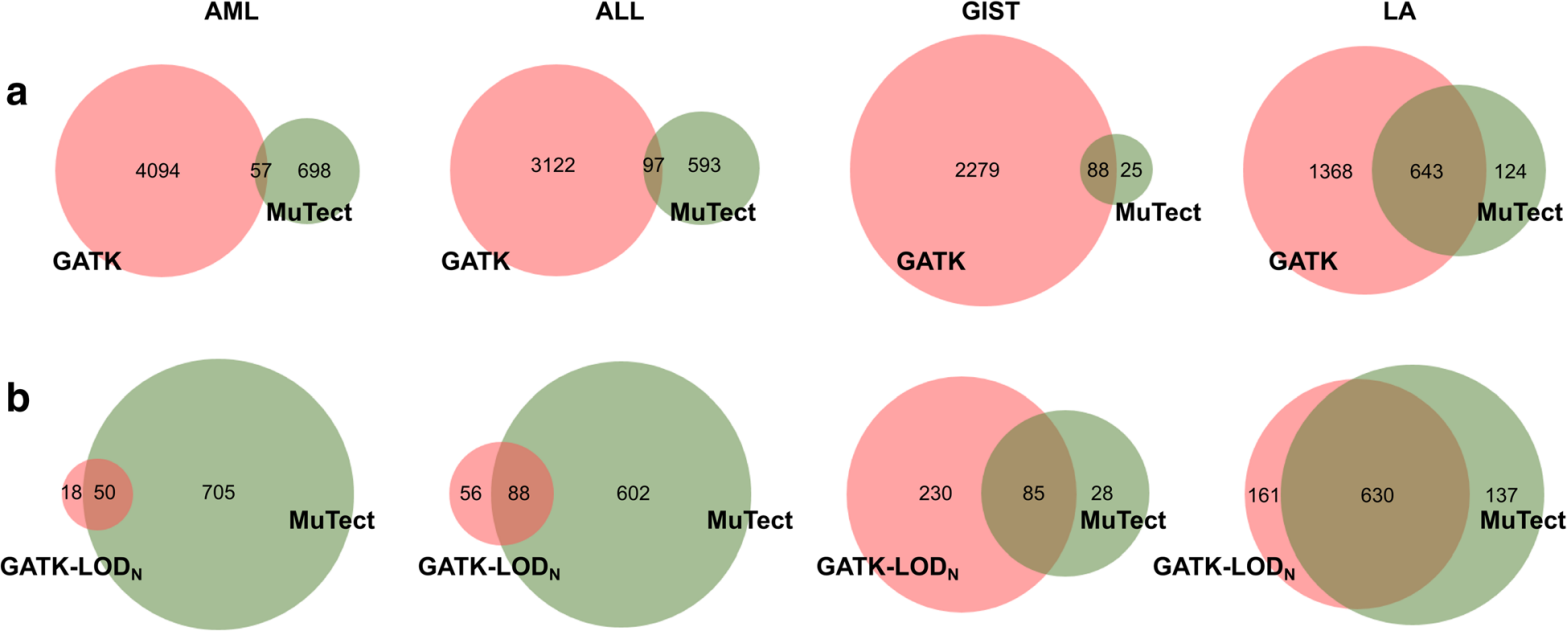
The GATK-LOD_N_ method reduces the number of GATK false positive calls. Comparison of the number of SNVs between GATK and MuTect before (**a**) and after (**b**) applying the GATK-LOD_N_ method for each cancer whole exome sequencing dataset. AML: Acute Myeloid Leukemia, ALL: Acute Lymphoblastic Leukemia, GIST: Gastrointestinal Stromal Tumor, LA: Lung Adenocarcinoma

We built a pipeline for discovery of single nucleotide variants (SNVs) in whole exome sequencing data and applied it to Acute Myeloid Leukemia (AML), Acute Lymphoid Leukemia (ALL), Gastrointestinal Stromal Tumor (GIST), and Lung Adenocarcinoma samples. First, we compared the results of the three variant calling procedures: MuTect, GATK, and GATK-LOD_N_. GATK detected 3 to 20 times more SNVs than MuTect (Fig. 2a) and the results for the Lung Adenocarcinoma dataset presented the highest concordance (30 %) between the two methods. GATK-LOD_N_ strongly reduced the number of SNVs in GATK results for the hematological tumors (Fig. 2b). For the solid tumors, approximately 10 % of GATK specific SNVs remained after applying GATK-LOD_N_, and, for the GIST dataset, it detected about three times more variants than MuTect.

The MuTect algorithm has two main parameters: Θ_T_ and Θ_N_. We calculated these values for a set of variants candidates (AML dataset) from GATK results and tested if we could reduce the number of false negatives by lowering these thresholds. We set the two parameters for Θ_T_ > = 4.5 and Θ_N|dbSNP site_ > = 3 and it permitted the detection of 10 variants previously profiled by Sanger sequencing, but not detected by the original MuTect analysis. However, the number of final candidates increased about 1.3 to 10 times in comparison with the original MuTect output (Table 2).

**Table 2:** Relaxing MuTect parameters increases the number of false positive calls. Number of variants found by MuTect, before and after relaxing the Θ_T_ and Θ_N_ parameters for six Acute Myeloid Leukemia (AML) normal-cancer sample pairs

Patients	MuTect	MuTect Adapted^a^
a1024	11	39
a1025	31	41
b1014	22	54
b2002	10	25
b2035	43	419
b2042	58	338

^a^Applying the computation of Θ_T_ and Θ_N_, from the MuTect algorithm, with lowered threshold values (4.5 and 3, respectively) downstream to the GATK analysis

We selected a set of candidate variants from the AML dataset and performed the validation experiment of each method in two rounds. In the first, we tested just the tumor samples, in order to evaluate the performance of each method in detecting the mutations. In the second round, we tested both tumor and normal samples, in order to evaluate the performance of each method in classifying mutations as somatic events. We observed that 18 out of 48 and 5 out of 18 GATK variants were correctly detected and classified, respectively, while MuTect presented high performance in both rounds (6 out of 7 and 2 out of 3, respectively). The GATK-LOD_N_ presented better validation rates than GATK for both mutation detection (18 out of 48 to 6 out of 9) and classification (5 out of 14 to 3 out of 4) (Table 3).

**Table 3:** The GATK-LOD_N_ method increases the GATK performance for both mutation detection and classification. The Sanger sequencing validation was performed in two rounds: in the first round we tested whether the methods correctly detected the mutation and in the second one we assessed whether the methods correctly classified the mutations as somatic events. The variant subsets tested (AML datatset) presented variants method specific and variants detected by one or more methods

	Mutation Detection^a^		Mutation Classification^b^	
	Tested	Validated	Tested	Validated
GATK-LOD_N_ - specific	4	1	2	2
GATK-LOD_N_ (All variants)	9	6	4	3
GATK (without LOD_N_) - specific	37	11	9	2
GATK (without LOD_N_) (All Variants)	48	18	14	5
MuTect - specific	22	21	8	8
MuTect (All Variants)	29	27	11	10
MuTect & GATK	7	6	3	2

^a^variants tested for correct mutation detection

^b^variants tested for correct classification as somatic events

Simulated data permitted the evaluation of sensitivity and specificity of the three variant calling methods. We measured the specificity by splitting a saliva sample alignment (80X) in two, applying to the pipeline and counting the number of called SNVs. Mutect, GATK, and GATK-LOD_N_ resulted in 8, 76 and 35 false positives, respectively. Then, we applied technical replicates of the same saliva sample to the pipeline and it resulted in 7, 84 and 33 false positives, respectively. We measured the sensitivity by simulating three artificial tumors with different Variant Allelic Frequency (VAF) ranges: one with high-frequency variants (*n* = 25, VAF: 0.97 to 1.0), one with intermediate-frequency variants (*n* = 25, VAF: 0.5 to 0.86), and another with low-frequency variants (*n* = 22, VAF: 0.02 to 0.25). MuTect presented a Positive Predictive Value (PPV) of 19/22 for low VAF mutations and its false negatives were composed by: one variant with VAF = 0.02, and two variants that had either VAF < 0.1 and total read depth smaller than 24 (Table 4). GATK presented the smallest performance for somatic variants, since it detected 2206 candidates out of 22 or 25 true positive variants. GATK-LOD_N_ presented a PPV of 17/22 for the low allelic frequency variants, but it missed variants with VAF < 0.095 (Table 4). MuTect detected all intermediate and high allelic frequency variants, while GATK-LOD_N_ presented PPVs of 23/30 and 23/31, respectively (Table 4).

**Table 4:** The GATK-LOD_N_ method presented good performance in artificial tumor samples. Performance of MuTect and GATK-LOD_N_ for artificial tumor samples that had variants with diverse allelic frequencies

		Artificial Tumor Samples		
		Low Frequency Variants (*n* = 22) VAF: 0.02 – 0.26	Intermediate Frequency Variants (*n* = 25) VAF: 0.5 – 0.86	High Frequency Variants (*n* = 25) VAF: 0.97 – 1
MuTect	Somatic Candidates	22	25	25
	TP	19	25	25
	FN	0	0	0
	FP	3	0	0
	PPV	19/22	25/25	25/25
	FDR	3/22	0/25	0/25
GATK-LOD_N_	Somatic Candidates	27	32	33
	TP	17	23	23
	FN	5	5	2
	FP	5	7	8
	PPV	17/22	23/30	23/31
	FDR	5/22	7/30	8/31

*TP* True positives, *FN* False negatives, *FP* False positives, *PPV* Positive Predictive Value (#TP / #FP + #TP), *FDR* False Discovery Rate (#FP / #FP + #TP), *VAF* Variant Allelic Frequency

GATK results were not reported in the table since it detected more than 2200 candidates out of 22 or 25 TPs

**Fig. 3:**
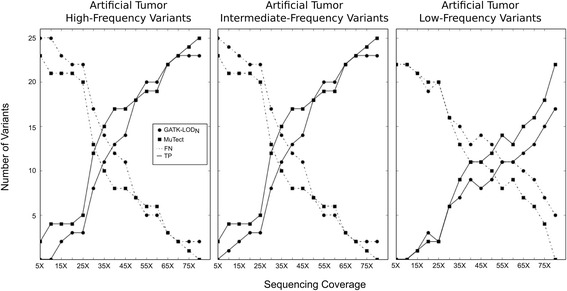
Number of False Negatives and True positives at different coverage levels. Three artificial tumors were created with 22, 25 and 25 SNVs, which had variant allelic fractions range of 0.02 to 0.25, 0.5 to 0.86, and 0.97 to 1.0, respectively. We counted the number of False Negatives (FN) and True positives (TP) for different levels of simulated sequencing coverage

For each artificial tumor, we simulated different sequencing coverages and evaluated the number of false negatives and true positives detected. We observed that, at different coverage levels, GATK-LOD_N_ and MuTect presented almost identical performance for the artificial tumors with high and intermediate variant frequency SNVs, except in the number of false negatives detected by GATK-LOD_N_ in the coverage interval of 5 to 20X. GATK-LOD_N_ presentedincreased number of detected true positives than MuTect in the coverage interval of 50 to 55X for high and intermediate-frequency variants, and in the coverage 20X for low-frequency variants (Fig. 3).

## Discussion

Our data show that the combination of standard tools - Genome Analysis Toolkit (GATK) and MuTect – improves the range of detected single nucleotide variants (SNVs) in whole exome sequencing data of cancer samples. We also developed the GATK-LOD_N_ method, which reduced the number of GATK false positive calls. Our study has the advantage of actually combining two different algorithms rather than proposing ways of unifying results of different tools [9, 20]. As one method originally presented high amounts of false positive calls (type I error) and the other high amounts of false negative calls (type II error), the GATK-LOD_N_ is an option of amplifying the range of detected SNVs without severely compromising sensitivity and specificity.

The GATK uses a Bayesian model to estimate the likelihood of a genotype given the observed sequence reads that cover the locus. It independently calls genotypes in tumor and normal samples, being the somatic mutations classified as those only present in the tumor sample. However, GATK detects many false positives likely due to germ-line variants with low sequencing coverage or low allelic frequency, that are not called in the normal samples. MuTect jointly analyzes tumor and normal samples, presenting high sensitivity, specificity and validation rates. Each method detects variants that the other does not detect, and a previous study demonstrated that the SNVs found only by GATK had relatively high validation rates [4]. One option would be taking into account just the results obtained from one tool, but it risks the selection of errors for which the algorithm is vulnerable [21]. Another option would be taking the intersection of multiple variant callers, but it will result in high false negative rates, since each tool uniquely identifies true variants [4]. We discarded the option of relaxing the MuTect parameters, since we observed that it included the detection of variants previously miscalled, but with the cost of including many false positives. Our study demonstrates the advantage of merging the results of MuTect and GATK-LOD_N_, since GATK-LOD_N_ reduces the number of GATK false positives and detect variants not detected by MuTect. The GATK-LOD_N_ increased the performance of GATK in the sequencing validation experiments and in the simulated artificial tumor samples. We observed that the GATK-LOD_N_ also outperformed MuTect in some simulated sequencing coverages. As sequencing datasets usually present large variability in coverage and quality, the different error modeling approaches and prior assumptions associated to the two methods should permit good performances in a wide scenario.

We performed the validation experiments just for variants from the hematological tumors (available in our laboratories), thus the validation rate might change for solid tumors. The results show that GATK-LOD_N_ filtered more variants in the hematological tumors than in the solid tumors and we hypothesized that the normal samples from hematological tumors may be more prone to contamination by cancer cells. Although GATK-LOD_N_ provided a small number of variants in the hematological datasets, even a single variant can give insights into the mechanisms of malignant transformation and help design personalized therapeutic approaches [22, 23]. We observed that the Lung Adenocarcinoma presented the biggest concordance between methods, maybe because patients with this type of cancer usually presents high mutation frequencies and harbors more somatic mutations compared with other cancer types [24–27]. The results also show that different methods may present bias to certain nucleotide substitution mutations, but more studies involving larger groups of tumors are needed.

The GATK-LOD_N_ is suitable for application together with other post-calling filtering features as: strand bias, nearby polymorphisms and technology specific sequencing errors removal [28–30]. For instance, Carson et al. [7] suggested new thresholds for genotype and variant filters to be used in conjunction with the GATK pipeline analysis, that could increase the GATK-LOD_N_ performance in population-based studies. Altogether, the GATK-LOD_N_ allows enough flexibility to deal with different study designs and requirements about how stringent the analysis must be.

Here, we presented a tested pipeline that combines standard tools, aiming to detect a wide range of somatic single nucleotide variants with high specificity and sensitivity. We developed the GATK-LOD_N_ method, which can be helpful in large-cohort discovery studies aimed to profile the somatic mutational landscape from whole exome sequencing data of cancer samples.

## Conclusion

Next generation sequencing analysis has drastically improved the biological knowledge of human cancers. Several tools and strategies are available to detect single nucleotide variants in normal-cancer paired samples, but many research groups report low concordance among them. In this study, we proposed a pipeline that applies two standard tools (MuTect and GATK) and one adapted method (GATK-LOD_N_) that increased the performance of its original algorithm. The GATK-LOD_N_ method improved the overall performance by reducing the number of false positive calls and permitted the detection of variants not detected by MuTect. We believe that the proposed pipeline will help in the understanding of cancer biology through the discovery of somatic single nucleotide variants in cancer sequencing data.

## Additional file

Additional file 1: Supplementary information. A.xls file including supplementary tables. (XLS 25 kb)

## Abbreviations

ALL: Acute lymphoblast leukemia
AML: Acute myeloid leukemia
FDR: False discovery rate
GATK: Genome analysis toolkit
GIST: Gastrointestinal stromal tumor
LA: Lung adenocarcinoma
MAF: Minor allelic frequency
PPV: Positive predictive value
SNVs: Single nucleotide variants
VAF: Variant allelic frequency
WES: Whole exome sequencing

## References

[1] Ding L, Wendl MC, Koboldt DC, Mardis ER (2010). Analysis of next-generation genomic data in cancer: accomplishments and challenges. Hum Mol Genet.

[2] Spencer DH, Tyagi M, Vallania F, Bredemeyer AJ, Pfeifer JD, Mitra RD, Duncavage EJ (2014). Performance of common analysis methods for detecting low-frequency single nucleotide variants in targeted next-generation sequence data. J Mol Diagn.

[3] Xu H, DiCarlo J, Satya RV, Peng Q, Wang Y (2014). Comparison of somatic mutation calling methods in amplicon and whole exome sequence data. BMC Genomics.

[4] O’Rawe J, Jiang T, Sun G, Wu Y, Wang W, Hu J, Bodily P, Tian L, Hakonarson H, Johnson WE, Wei Z, Wang K, Lyon GJ (2013). Low concordance of multiple variant-calling pipelines: practical implications for exome and genome sequencing. Genome Med.

[5] Liu B, Morrison CD, Johnson CS, Trump DL, Qin M, Conroy JC, Wang J, Liu S (2013). Computational methods for detecting copy number variations in cancer genome using next generation sequencing: principles and challenges. Oncotarget.

[6] Pabinger S, Dander A, Fischer M, Snajder R, Sperk M, Efremova M, Krabichler B, Speicher MR, Zschocke J, Trajanoski Z (2014). A survey of tools for variant analysis of next-generation genome sequencing data. Brief Bioinform.

[7] Carson AR, Smith EN, Matsui H, Brækkan SK, Jepsen K, Hansen J-B, Frazer KA (2014). Effective filtering strategies to improve data quality from population-based whole exome sequencing studies. BMC Bioinformatics.

[8] Bodini M, Ronchini C, Giac L, Russo A, Melloni GEM, Luzi L, Sardella D, Volorio S, Hasan SK, Ottone T, Lavorgna S, Lo-coco F, Candoni A, Fanin R, Toffoletti E, Iacobucci I, Martinelli G, Cignetti A, Tarella C, Bernard L, Pelicci PG, Riva L (2015). Perspectives the hidden genomic landscape of acute myeloid leukemia : subclonal structure revealed by undetected mutations. Blood.

[9] Kim SY, Jacob L, Speed TP (2014). Combining calls from multiple somatic mutation-callers. BMC Bioinformatics.

[10] Kang G, Yun H, Sun C, Park I, Kwon J, Do I, Hong ME, Van Vrancken M, Park JO, Cho J, Kim K, Sohn TS (2016). Integrated genomic analyses identify frequent gene fusion events and VHL inactivation in gastrointestinal stromal tumors. Oncotarget.

[11] Seo JS, Ju YS, Lee WC, Shin JY, Lee JK, Bleazard T, Lee J, Jung YJ, Kim JO, Shin JY, Yu SB, Kim J, Lee ER, Kang CH, Park IK, Rhee H, Lee SH, Kim JI, Kang JH, Kim YT (2012). The transcriptional landscape and mutational profile of lung adenocarcinoma. Genome Res.

[12] FASTX-Toolkit http://hannonlab.cshl.edu/fastx_toolkit/.

[13] Li H, Durbin R (2009). Fast and accurate short read alignment with burrows-wheeler transform. Bioinformatics.

[14] Picard Tools http://broadinstitute.github.io/picard/.

[15] DePristo MA, Banks E, Poplin R, Garimella KV, Maguire JR, Hartl C, Philippakis AA, del Angel G, Rivas MA, Hanna M, McKenna A, Fennell TJ, Kernytsky AM, Sivachenko AY, Cibulskis K, Gabriel SB, Altshuler D, Daly MJ (2011). A framework for variation discovery and genotyping using next-generation DNA sequencing data. Nat Genet.

[16] Cibulskis K, Lawrence MS, Carter SL, Sivachenko A, Jaffe D, Sougnez C, Gabriel S, Meyerson M, Lander ES, Getz G (2013). Sensitive detection of somatic point mutations in impure and heterogeneous cancer samples. Nat Biotechnol.

[17] Wang K, Li M, Hakonarson H (2010). ANNOVAR: functional annotation of genetic variants from high-throughput sequencing data. Nucleic Acids Res.

[18] Breese MR, Liu Y (2013). NGSUtils: a software suite for analyzing and manipulating next-generation sequencing datasets. Bioinformatics.

[19] Hansen NF, Gartner JJ, Mei L, Samuels Y, Mullikin JC (2013). Shimmer: detection of genetic alterations in tumors using next-generation sequence data. Bioinformatics.

[20] Hansen MC, Nederby L, Roug A, Villesen P, Kjeldsen E, Nyvold CG, Hokland P (2015). Novel scripts for improved annotation and selection of variants from whole exome sequencing in cancer research. MethodsX.

[21] Roberts ND, Kortschak RD, Parker WT, Schreiber AW, Branford S, Scott HS, Glonek G, Adelson DL (2013). A comparative analysis of algorithms for somatic SNV detection in cancer. Bioinformatics.

[22] Lyon GJ, Wang K (2012). Identifying disease mutations in genomic medicine settings: current challenges and how to accelerate progress. Genome Med.

[23] Ciriello G, Miller ML, Aksoy BA, Senbabaoglu Y, Schultz N, Sander C (2013). Emerging landscape of oncogenic signatures across human cancers. Nat Genet.

[24] Kim N, Hong Y, Kwon D, Yoon S (2013). Somatic mutaome profile in human cancer tissues. Genomics Inform.

[25] Lawrence MS, Stojanov P, Polak P, Kryukov GV, Cibulskis K, Sivachenko A, Carter SL, Stewart C, Mermel CH, Roberts SA, Kiezun A, Hammerman PS, McKenna A, Drier Y, Zou L, Ramos AH, Pugh TJ, Stransky N, Helman E, Kim J, Sougnez C, Ambrogio L, Nickerson E, Shefler E, Cortés ML, Auclair D, Saksena G, Voet D, Noble M, DiCara D (2013). Mutational heterogeneity in cancer and the search for new cancer-associated genes. Nature.

[26] Kandoth C, McLellan MD, Vandin F, Ye K, Niu B, Lu C, Xie M, Zhang Q, McMichael JF, Wyczalkowski MA, Leiserson MDM, Miller CA, Welch JS, Walter MJ, Wendl MC, Ley TJ, Wilson RK, Raphael BJ, Ding L (2013). Mutational landscape and significance across 12 major cancer types. Nature.

[27] Collisson EA, Campbell JD, Brooks AN, Berger AH, Lee W, Chmielecki J, Beer DG, Cope L, Creighton CJ, Danilova L, Ding L, Getz G, Hammerman PS, Neil Hayes D, Hernandez B, Herman JG, Heymach JV, Jurisica I, Kucherlapati R, Kwiatkowski D, Ladanyi M, Robertson G, Schultz N, Shen R, Sinha R, Sougnez C, Tsao M-S, Travis WD, Weinstein JN, Wigle DA (2014). Comprehensive molecular profiling of lung adenocarcinom. Nature.

[28] Reumers J, De Rijk P, Zhao H, Liekens A, Smeets D, Cleary J, Van Loo P, Van Den Bossche M, Catthoor K, Sabbe B, Despierre E, Vergote I, Hilbush B, Lambrechts D, Del-Favero J (2011). Optimized filtering reduces the error rate in detecting genomic variants by short-read sequencing. Nat Biotechnol.

[29] Nakamura K, Oshima T, Morimoto T, Ikeda S, Yoshikawa H, Shiwa Y, Ishikawa S, Linak MC, Hirai A, Takahashi H, Altaf-Ul-Amin M, Ogasawara N, Kanaya S (2011). Sequence-specific error profile of illumina sequencers. Nucleic Acids Res.

[30] Quail MA, Smith M, Coupland P, Otto TD, Harris SR, Connor TR, Bertoni A, Swerdlow HP, Gu Y (2012). A tale of three next generation sequencing platforms: comparison of Ion torrent, pacific biosciences and illumina MiSeq sequencers. BMC Genomics.

